# Serum HE4 concentration differentiates malignant ovarian tumours from ovarian endometriotic cysts

**DOI:** 10.1038/sj.bjc.6605011

**Published:** 2009-03-31

**Authors:** K Huhtinen, P Suvitie, J Hiissa, J Junnila, J Huvila, H Kujari, M Setälä, P Härkki, J Jalkanen, J Fraser, J Mäkinen, A Auranen, M Poutanen, A Perheentupa

**Affiliations:** 1 Department of Physiology, Institute of Biomedicine, University of Turku, FI-20520 Turku, Finland; 2 Department of Obstetrics and Gynaecology, Turku University Hospital, FI-20520 Turku, Finland; 3 Department of Mathematics, University of Turku, FI-20014 Turku, Finland; 4 Department of Pathology, Turku University Hospital, FI-20520 Turku, Finland; 5 Department of Obstetrics and Gynaecology, Päijät-Häme Central Hospital, FI-15850 Lahti, Finland; 6 Department of Obstetrics and Gynaecology, Helsinki University Hospital, FI-00029 Helsinki, Finland; 7 Department of Obstetrics and Gynaecology, North Karelia Central Hospital, FI-80210 Joensuu, Finland; 8 Turku Center for Disease Modeling, University of Turku, FI-20520 Turku, Finland

**Keywords:** HE4, CA125, endometriosis, ovarian cancer

## Abstract

Human epididymal secretory protein E4 (HE4, also known as WAP four-disulphide core domain protein 2) is a new promising biomarker for ovarian cancer but its specificity against ovarian endometriotic cysts is only superficially known. We, thus, analysed serum HE4 concentrations together with a tumour marker CA125 in serum samples of women diagnosed with various types of endometriosis, endometrial cancer or ovarian cancer, and in samples from healthy controls. The mean serum concentration of HE4 was significantly higher in serum samples of patients with both endometrial (99.2 pM, *P*<0.001) and ovarian (1125.4 pM, *P*<0.001) cancer but not with ovarian endometriomas (46.0 pM) or other types of endometriosis (45.5 pM) as compared with healthy controls (40.5 pM). The serum CA125 concentrations were elevated in patients with ovarian cancer, advanced endometriosis with peritoneal or deep lesions, or ovarian endometriomas, but not in the patients with endometrial cancer. The microarray results revealed that the mRNA expression of the genes encoding HE4 and CA125 reflected the serum protein concentrations. Taken together, measuring both HE4 and CA125 serum concentrations increases the accuracy of ovarian cancer diagnosis and provides valuable information to discriminate ovarian tumours from ovarian endometriotic cysts.

Endometriosis is one of the most common benign gynaecological conditions, it may affect up to 10% of women in reproductive age. The typical symptoms caused by endometriosis are pain and subfertility. Endometriosis is characterised by the appearance of endometrial glands and stroma in ectopic locations outside the uterine cavity. The lesions are typically located on the peritoneum, in the ovaries or infiltered into the organs within the abdominal cavity (Giudice and Kao, 2004). There are several theories for the aetiology of endometriosis and it has been suggested that the peritoneal lesions, deep rectovaginal lesions, and ovarian endometriotic cysts called endometriomas would be of different origin (Nisolle and Donnez, 1997).

Although transvaginal ultrasound examination is of value, particularly in women with ovarian endometriomas, the diagnosis of endometriosis typically requires laparoscopic verification. The ultrasound is not a useful tool in the diagnostics of peritoneal implants and adhesions or in staging of the disease. In addition, neoplastic ovarian cysts can resemble endometriomas in ultrasound and, therefore, need to be carefully considered in the differential diagnostics. Furthermore, endometriosis is shown to increase the risk of certain subtypes of ovarian cancer, such as endometrioid and clear-cell carcinomas (Nagle *et al*, 2008). There are data indicating that 40% of endometrioid ovarian carcinomas and 50% of clear-cell ovarian carcinomas are associated with endometriosis (Sato *et al*, 2000). Both endometrioid and clear-cell carcinomas are thought to arise, at least partly, from endometriosis. Similar pathophysiological mechanisms may be involved in the progression of endometriosis as well as in its transformation into ovarian neoplasia (Ness, 2003).

Currently, CA125 antigen is the most commonly used biochemical marker in ovarian cancer diagnostics. However, it is associated with a high false-positive rate among women with benign gynaecological conditions such as endometriosis (Markman, 1997). Furthermore, CA125 has very low sensitivity in identifying patients with early-stage ovarian cancer (Terry *et al*, 2004). Thus, to improve the specificity and sensitivity of ovarian cancer diagnosis, the use of novel biomarkers such as HE4 (human epididymal secretory protein E4; WAP four-disulphide core domain protein 2, WFDC2) alone or in combination with CA125 has been intensively studied (Hellström *et al*, 2003; Havrilesky *et al*, 2008; Moore *et al*, 2008a).

In addition to diagnosis of ovarian cancer, CA125 can be used to evaluate the efficacy of endometriosis therapy as well as the recurrence of endometriosis. However, the lack of sensitivity and specificity of the biomarker has significantly hampered its use as a diagnostic test (Mol *et al*, 1998; Kitawaki *et al*, 2005). On the other hand, a panel of markers including CA125 has been suggested to predict the presence of endometriosis in a subset of patients (Seeber *et al*, 2008).

Human epididymal secretory protein E4 is a new serological biomarker for diagnosis of ovarian cancer. As compared with the markers used previously, it possesses increased sensitivity for detecting ovarian cancer, especially the stage I disease (Havrilesky *et al*, 2008; Moore *et al*, 2008a), whereas the HE4 encoding gene WFDC2 is expressed particularly in serous and endometrioid ovarian cancer (Drapkin *et al*, 2005; Galgano *et al*, 2006). However, it is shown to be expressed also in some other types of tumours, for example lung adenocarcinoma (Galgano *et al*, 2006). Its expression is also apparent in normal endometrial glands and endometrial cancer (Drapkin *et al*, 2005; Galgano *et al*, 2006), but the expression in endometriotic lesions including ovarian endometriomas is not known.

In the present study, we measured serum concentrations of HE4 and CA125 in 129 patients with endometriosis of whom 69 had ovarian endometriomas. The data were compared with results obtained in 16 patients with endometrial cancer, 14 with ovarian cancer, and 66 healthy controls. The mRNA expression of genes encoding HE4 and CA125, namely WFDC2 and MUC16, respectively, was also analysed in the tissue specimens of various types of endometriosis, ovarian cancer, endometrial cancer, and normal endometrium.

## Patients and methods

### Patients

The patients were enrolled into the study in two Central Hospitals and two University Central Hospitals in Finland between October 2005 and November 2007. A written informed consent was required from all patients before sampling, and the study protocol was approved by the joint ethics committee of Turku University and Turku University Central Hospital, Turku, Finland.

The serum samples of women diagnosed with endometriosis (Endo, *n*=129), ovarian cancer (OvCa, *n*=14), or endometrial cancer (EmCa, *n*=16) were included into the study, together with 66 samples collected from healthy controls (Ctrl). The serum samples of patients with ovarian endometrioma (OvEndo, *n*=69, ASRM stage 3–4) were evaluated as a separate group in the analysis. The diseases were diagnosed per operatively in laparoscopy or laparotomy and confirmed by histopathological evaluation. Description of patients included in the study is presented in Table 1. Patients with endometriosis were classified to stage 1–4 according to the revised American Society for Reproductive Medicine (ASRM) criteria (1997). The patients with ovarian and endometrial cancer were staged according to the FIGO guidelines (Benedet *et al*, 2000). The 14 ovarian carcinomas included 7 serous, 3 mucinous, 2 clear-cell, 1 endometrioid, and 1 small-cell carcinomas. Four of the ovarian cancers were local stage I cancers and the remaining 10 were of advanced stage II–IV. All endometrial carcinomas were endometrioid adenocarcinomas. In 14 patients the cancer was limited to the uterus (stage I–II), whereas in 2 cases metastatic pelvic lymph nodes were present (stage III). Control subjects (*n*=66) were verified to be free from endometriosis or ovarian cancer by laparoscopy during the tubal sterilisation, and the possibility of endometrial cancer was excluded by endometrial biopsy. The mean age of patients with endometriosis, ovarian cancer, endometrial cancer, and healthy controls was 31.8, 63.8, 60.5, and 38.5 years, respectively.

The gene expression of WFDC2 and MUC16, which encode the HE4 and CA125 proteins, respectively, was evaluated in 149 non-ovarian endometriotic lesions, 28 ovarian endometriotic cysts, 15 ovarian and 14 endometrium carcinomas, 64 individual endometrium samples from patients with endometriosis, and 41 endometrium samples from healthy controls. Thirteen of the ovarian carcinomas were serous, one was endometrioid and one undifferentiated. All endometrial carcinomas were endometrioid adenocarcinomas. The tissue samples were collected during laparoscopic or open surgery. Endometrial biopsies were collected using a sterile Pipelle sampler (Pipelle de Cornier; Laboratoire CCD, Paris, France). All tissue samples were snap-frozen and stored in liquid nitrogen.

### Serum HE4 and CA125 analysis

Serum samples were collected just before surgery into non-heparinised tubes and centrifuged for 15 min at 3000 r.p.m. (800 *g*) after keeping 30 min at room temperature. The serum was stored at −20 or −80°C. Human epididymal secretory protein E4 and CA125 concentrations were analysed in serum samples by ELISA analysis (Fujirebio Diagnostics Inc., Malvern, PA, USA) according to manufacturer's instructions.

### Expression analysis

The gene expression levels of WFDC2 and MUC16 were studied as part of our whole-genome microarray analysis. The total RNA was isolated with Trizol reagent (Invitrogen, Carlsbad, CA, USA), further purified with RNeasy columns (Qiagen, Valencia, CA, USA) and DNase treated (RNase-free DNase Set (Qiagen) or DNase I (Invitrogen)). The RNA concentrations were measured with NanoDrop ND-1000 (Thermo Fisher Scientific, Waltham, MA, USA) and RNA quality was controlled by Experion analysis (Bio-Rad Laboratories, Hercules, CA, USA). All subsequent steps of the microarray analysis were carried out at the Finnish DNA-Microarray Centre utilising the Sentrix Human Illumina 6 V2 Expression BeadChips (Illumina, San Diego, CA, USA), which contains over 47 000 known genes, gene candidates, and splice variants. RNA sample (300 ng each) was used as template for producing double-stranded cDNA, and then biotinylated cRNA using the Illumina RNA TotalPrep Amplification Kit (Ambion Inc., Austin, TX, USA). The labelled cRNA was purified and hybridised to the BeadChip at 55°C for 16 h following the Illumina Whole-Genome Gene Expression Protocol for BeadStation. Hybridisation was detected with Cyanine3-streptavidine (GE Healthcare, Little Chalfont, UK) and the arrays were scanned with the Illumina BeadArray Reader. Normalisation and statistical analyses of the microarray data were performed using the statistical software R package limma (http://www.R-project.org) or Sigma Stat 3.1 (SPSS Inc., Chicago, IL, USA). The expression levels were analysed using the probes ILMN_1706612 and ILMN_1799120 for WFDC2 and ILMN_1736316 for MUC16.

### Statistical analyses

The statistical analyses of serum HE4 and CA125 concentrations alone and in combination were performed using Tukey's multiple comparisons of means with 95% family-wise confidence level.

The classification capability of the HE4 and CA125 markers, alone and together, was assessed using the binary and multinomial logistic regression models with leave-one-out cross-validation. In the cross-validation each sample in turn was reserved for testing whereas the others were used in the model building. The sensitivity at 95% specificity and the proportion of correctly classified samples (accuracy) was calculated for each cross-validated regression model. Also, for each binary model, the receiver operator characteristic curves were constructed and the area under the curve was used to summarise the overall performance of the regression model.

## Results

### Serum HE4 and CA125 concentrations

**Figure 1:**
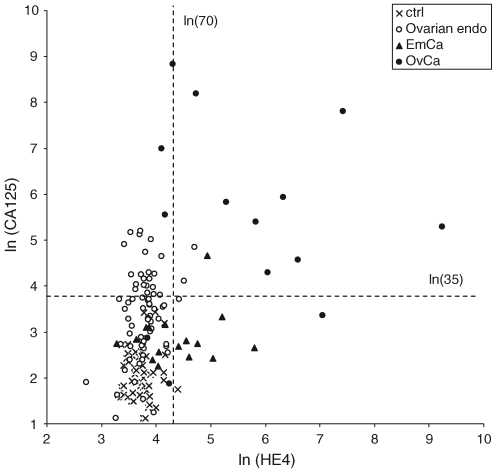
Correlation between serum HE4 (pM) and CA125 (U ml^−1^) concentrations as ln-transformation in patients with ovarian endometriosis (Ovarian endo, **○**), ovarian cancer (OvCa, **•**), endometrial cancer (EmCa, **▴**), and controls (ctrl, X). The broken lines show the threshold values for positive diagnosis of CA125 (35 U ml^−1^) and HE4 (70 pM, according to Moore *et al* (2008a)).

The mean serum HE4 concentrations were similar and below the 70 pM limit for elevated value (Moore *et al*, 2008a) in patients with endometriosis (mean 45.5 pM) and in healthy controls (40.5 pM), irrespective of the disease classification or the presence of ovarian endometrioma (ASRM stage 1: 46.7 pM; stage 2: 44.9 pM; stage 3: 43.2 pM; stage 4: 46.5 pM; OvEndo: 46.0 pM). However, in addition to the highly increased HE4 concentration in patients with ovarian cancer (1125.4 pM), the HE4 serum concentration was significantly elevated also in patients with endometrial cancer (99.2 pM, *P*<0.001). The levels of HE4 in different types of ovarian cancer were highest in serous (2031.1 pM, *n*=7) carcinomas, whereas it was clearly elevated also in clear-cell (397.6 pM, *n*=2) and mucinous (202.6 pM, *n*=3) carcinomas. The serum concentrations in the different patient groups are presented in Table 2 and Figure 1.

The serum levels of CA125 were highest in patients with ovarian cancer (mean 1117.1 U ml^−1^, *P*<0.001) but were also significantly (*P*<0.001) elevated in patients with ovarian endometrioma (44.3 U ml^−1^) and advanced non-ovarian endometriosis (ASRM stage 4, 40.8 U ml^−1^) as compared with healthy controls (8.9 U ml^−1^). These concentrations were also higher than the threshold value for elevated CA125 result (35 U ml^−1^). The concentration increased with increasing ASRM stage of endometriosis (Table 2). However, the median concentration (33.7 U ml^−1^) in patients with endometrioma is below the threshold value for elevated CA125 result. In sera of patients with endometrial cancer the level of CA125 (22.0 U ml^−1^) was also significantly (*P*=0.029) higher than in healthy controls even though clearly lower than the threshold value.

Sensitivities for the separation of the patient groups by HE4, CA125, or their combination were calculated for each two-wise comparison at specificity of 95%. To differentiate the patients with ovarian cancer from healthy controls, the combination of CA125 and HE4 relative to CA125 or HE4 alone resulted in the highest accuracy (96.3%) and sensitivity (92.9%; Table 3). Furthermore, the combination had the highest accuracy (94.0%) and sensitivity (78.6%) also for differential diagnosis of patients with ovarian cancer from those with ovarian endometriosis. The combination also differentiates ovarian endometriosis from healthy controls almost as accurately as CA125 alone, even though HE4 alone is a poor marker for endometriosis. Finally, the combination of HE4 and CA125 had the highest accuracy (81.9%) also in the three-wise comparison between the ovarian cancer, ovarian endometriosis, and healthy controls.

### Expression of HE4 and CA125 encoding genes

The mRNA expression of WFDC2 (encoding HE4) and MUC16 (encoding CA125) in the tissue samples is shown in Table 4. The expression in ovarian cancer was compared to that of ovarian endometrioma, and the expression in endometrial cancer and non-ovarian endometriotic lesions was compared with that of the normal endometrium of healthy controls. The expression of WFDC2 was significantly (*P*<0.05) higher in ovarian cancer (median of log2 intensity value 9.25) than in the ovarian endometrioma (6.73) with fold change (FC) of 5.7. However, the 1.9-fold higher expression in endometrial cancer (8.61) did not reach significance when compared with healthy endometrium (7.67). The expression data is, thus, in line with the differential serum concentrations observed for HE4 in the different patient groups and controls. In contrast, the mRNA expression of MUC16 was relatively stable in the various specimens, whereas the FC between all comparisons was between 0.7 and 1.3.

## Discussion

HE4 is a novel serological marker used especially for ovarian cancer diagnosis (Hellström *et al*, 2003; Gagnon and Ye, 2008; Hellström and Hellström, 2008; Moore *et al*, 2008a). Because of its high sensitivity, it is useful also for detecting stage I ovarian cancer (Havrilesky *et al*, 2008; Moore *et al*, 2008a). Furthermore, HE4 has been suggested as a biomarker for the diagnosis of endometrial cancer (Moore *et al*, 2008b). Currently, several biomarker panels are being evaluated to increase the sensitivity and specificity of ovarian cancer diagnosis. The combination of CA125 and HE4 with, or without, other biomarkers such as Glycodelin, Plau-R, MUC-1, PAI-1 (Havrilesky *et al*, 2008), SMRP (Hellström and Hellström, 2008; Moore *et al*, 2008a), CA72-4, and osteopontin (Moore *et al*, 2008a) has been evaluated to improve ovarian cancer diagnosis. The data suggest that by combining these markers the predictive accuracy in ovarian malignancy is better than by applying any of the markers alone. The panel of biomarkers including HE4 has been evaluated also for monitoring the recurrence of ovarian cancer (Havrilesky *et al*, 2008; Moore *et al*, 2009).

In female tissues, HE4 immunoreactivity has been shown to be highest in glandular epithelium of the genital tract, including endocervical glands, endometrial glands, fallopian tubes, and Bartholin's glands (Drapkin *et al*, 2005; Galgano *et al*, 2006). In contrast to the normal ovarian surface epithelium, which does not express HE4, cortical inclusion cysts lined by metaplastic Müllerian epithelium have been shown to express the protein abundantly (Drapkin *et al*, 2005). The expression of HE4 protein in ovarian tumours is highest in serous carcinomas but immunostaining has been detected also in the vast majority of ovarian endometrioid and clear-cell carcinomas (Drapkin *et al*, 2005; Galgano *et al*, 2006). In addition to ovarian carcinoma, some pulmonary, endometrial, and breast adenocarcinomas have been shown to express HE4 (Galgano *et al*, 2006). Although the protein has been detected in both normal and malignant endometrium, the expression of HE4 in the endometriotic lesions is only superficially known. Recently, Moore *et al* (2008a) analysed HE4 and eight other biomarkers in the sera of 166 patients with ovarian cancer or with several other kinds of pelvic masses, of whom 29 had endometriosis. They showed that the HE4 and CA125 concentrations were the best combination of biomarkers to distinguish ovarian cancer patients from those with other pelvic masses. However, the types of endometriosis lesions in these patients were not described.

In agreement with other recent studies (Hellström *et al*, 2003; Gagnon and Ye, 2008; Hellström and Hellström, 2008; Moore *et al*, 2008a, 2008b), we detected increased HE4 concentration in patients with ovarian and endometrial cancer. The present data demonstrate that neither the expression of HE4 encoding gene in the endometriotic lesions nor serum HE4 concentration in the patients with endometriosis with any types of endometriosis is increased. It is of specific interest to note that HE4 is not increased even in patients with ovarian endometriosis. In contrast, the serum level of CA125 was increased in patients with advanced endometriosis and ovarian endometriomas, as expected. It should be noted that endometriosis is typically diagnosed at young adult age (25–35 years) and often disappears after menopause, whereas the incidence of ovarian cancer increases in older women (highest incidence at the age of 50–60 years). Interestingly, it has been reported that the concentration of HE4 increases with age in healthy postmenopausal woman whereas CA125 does not (Lowe *et al*, 2008).

Thus, measuring both HE4 and CA125 together, rather than either of them alone, provides a more accurate tool for differential diagnosis of patients with ovarian cancer and ovarian endometriotic cysts from healthy subjects. It may also help clinicians in the follow-up of patients suffering from advanced endometriosis when considering the possibility of malignant transformation of the lesions. Within the patients with ultrasound-detected ovarian mass, the high serum HE4 with high CA125 would suggest the presence of ovarian cancer whereas elevated CA125 without elevated HE4 would direct towards advanced or ovarian endometrioma or other benign conditions. Furthermore, the elevated serum HE4 concentration with normal CA125 concentration would suggest the presence of either ovarian or possibly other type of cancer, for example endometrial cancer.

The greatest benefit of highly specific differentiation between ovarian cancer and endometriosis may well be found in identification of ovarian cancer in the early non-symptomatic stage. A high proportion of ovarian cancers are diagnosed at an advanced stage with a dismal survival rate. In contrast, the 5-year survival rate for stage I disease with the malignancy confined to the ovary is above 90%. This emphasises the importance of detecting the ovarian cancers at their early stage to improve the mortality rate.

In summary, the serum concentration of HE4, a novel biomarker for ovarian cancer, was not increased in patients with ovarian endometrioma or any other types of endometriosis, whereas the serum CA125 concentration was increased in patients with advanced endometriosis. The results, thus, suggest that the serum HE4 concentration is a valuable marker to better distinguishing patients with ovarian malignancies from those suffering from the benign ovarian endometriotic cysts.

## Figures and Tables

**Table 1:** Description of patients included in the analysis of serum HE4 and CA125

		**Classification (ASRM)/*stage (FIGO)***			
**Diagnosis**	***n***	**1/*I***	**2/*II***	**3/*III***	**4/*VI***
Healthy	66				
					
*Endometriosis all*	129	16	17	33	63
Ovarian endometrioma	69	—	—	24	45
Endometrial cancer	16	*13*	*1*	*2*	—
					
*Ovarian cancer*	14	*4*	*1*	*7*	*2*
Serous	7	—	—	*6*	*1*
Mucinous	3	3	—	—	—
Clear cell	2	—	*1*	*1*	—
Endometrioid	1	1	—	—	—
Small cell	1	—	—	—	*1*
Total	225				

**Table 2:** Serum HE4 and CA125 concentrations in patients with endometriosis, endometrial, and ovarian cancer

		**HE4 (pM)**					**CA125 (U ml^−1^)**				
**Diagnosis**	***n***	**Mean**	**s.d.**	**Median**	**(range)**	***P*-value^a^**	**Mean**	**s.d.**	**Median**	**(range)**	***P*-value^a^**
*Controls*	66	40.5	10.3	38.6	(27.0–80.7)	—	8.9	6.2	6.7	(2.2–31.2)	—
											
*Endometriosis all*	129	45.5	13.4	43.5	(15.2–111.0)	—	35.8	36.0	25.3	(0.8–182.0)	—
Stage 1	16	46.7	11.4	42.9	(35.1–80.9)	0.96192	15.6	9.8	11.5	(5.1–44.4)	0.34560
Stage 2	17	44.9	13.1	43.9	(28.7–72.0)	0.99718	21.4	18.4	14.8	(3.5–70.5)	0.09637
Stage 3	33	43.2	11.4	43.4	(15.2–68.0)	—	25.1	22.7	14.8	(0.9–79.0)	—
Stage 3 w/o OvEndo	9	43.9	10.1	43.8	(32.6–61.9)	0.99971	23.3	25.4	13.4	(2.6–79.0)	0.58802
Stage 4	63	46.5	15.0	44.0	(26.8–111.0)	—	50.4	43.1	36.7	(0.8–182.0)	—
Stage 4 w/o OvEndo	18	43.5	12.1	40.9	(27.4–64.5)	0.99973	40.8	30.9	32.1	(0.8–127.0)	0.00001
Ovarian endometriosis	69	46.0	14.9	44.0	(15.2–111.0)	0.89441	44.3	42.1	33.7	(0.9–182.0)	0.00000
Endometrial cancer	16	99.2	76.4	73.3	(26.5–330.5)	0.00001	22.0	23.0	15.5	(9.6–106.0)	0.02915
											
*Ovarian cancer*	14	1125.4	2670.0	268.3	(46.5–10250.0)	0.00000	1117.1	1971.0	240.0	(6.6–6890.0)	0.00000
						0.00000^b^					0.00000^b^
Serous	7	2031.1	3669.1	562.5	(46.5–10250.0)	—	1938.7	2588.7	341.0	(28.9–6890.0)	—
Mucinous	3	202.6	189.7	113.0	(74.4–420.5)	—	201.5	186.0	221.0	(6.6–377.0)	—
Clear cell	2	397.6	477.2	397.6	(60.1–735.0)	—	674.5	587.6	674.5	(259.0–1090.0)	—
Endometrioid	1	70.0	—	70.0	—	—	97.0	—	97.0	—	—
Small cell	1	64.1	—	64.1	—	—	17.8	—	17.8	—	—

a Tukey's multiple comparisons of means, in comparison to the healthy controls.

b Comparison to patients with ovarian endometriosis.

**Table 3:** Tumour marker accuracy and sensitivity at 95% specificity for ovarian cancer and ovarian endometriosis

**Markers**	**Accuracy (%)**	**ROC-AUC (%)**	**Sensitivity (%)**
*OvCa vs OvEndo*			
CA125+HE4	94.0	91.3	78.6
CA125	92.8	77.0	64.3
HE4	91.6	91.9	71.4
			
*OvCa vs ctrl*			
CA125+HE4	96.3	91.1	92.9
CA125	96.3	91.7	78.6
HE4	93.8	95.5	78.6
			
*OvEndo vs ctrl*			
CA125+HE4	82.2	86.8	62.3
CA125	83.0	87.7	60.9
HE4	60.7	60.5	5.8
			
*OvCa vs OvEndo vs ctrl*			
CA125+HE4	81.9		
CA125	81.2		
HE4	59.7		

OvCa=ovarian cancer; OvEndo=ovarian endometriosis.

**Table 4:** Expression of WFDC2 (HE4) and MUC16 (CA125) mRNA in tissue specimens of endometrium, non-ovarian and ovarian endometriosis, endometrial cancer, and ovarian cancer as log2 intensity values

		**WFDC2**					**MUC16**				
**Sample group**	***N***	**Median**	**25%**	**75%**	**FC**	***P*-value^a^**	**Median**	**25%**	**75%**	**FC**	***P*-value^a^**
E of healthy control	41	7.67	6.96	8.28			7.1	6.76	7.48		
E of endo patient	64	7.75	7.17	8.28	1.1^b^	NS^b^	7.21	6.86	7.63	1.1^b^	NS^b^
Non-ovarian endo	149	7.03	6.65	7.44	0.6^b^	<0.05^b^	6.62	6.48	6.84	0.7^b^	<0.05^b^
Ovarian endo	28	6.73	6.45	7.13			6.49	6.37	6.65		
EmCa	14	8.61	8.06	9.24	1.9^b^	NS	6.28	6.14	7.29	0.6^b^	<0.05^b^
OvCa	15	9.25	8.36	10.04	5.7^c^	<0.05^c^	6.86	6.66	7.74	1.3^c^	<0.05^c^

E=endometrium; EmCA=endometrial cancer; endo=endometriosis; OvCa=ovarian cancer; NS=not significant.

a Dunn's method.

b Compared to endometrium specimens of healthy controls.

c Compared to ovarian endometriomas.
